# Anal Sphincter Augmentation Using Biological Material

**DOI:** 10.3389/fsurg.2015.00060

**Published:** 2015-11-24

**Authors:** Nasra N. Alam, Sunil K. Narang, Ferdinand Köckerling, Ian R. Daniels, Neil J. Smart

**Affiliations:** ^1^Exeter Surgical Health Services Research Unit (HeSRU), Royal Devon and Exeter Hospital, Exeter, UK; ^2^Department of Surgery, Center for Minimally Invasive Surgery, Academic Teaching Hospital of Charité Medical School, Vivantes Hospital, Berlin, Germany

**Keywords:** fecal incontinence, anal sphincter repair, overlapping sphincter repair, anal encirclement, anal bulking, biological material

## Abstract

**Introduction:**

The aim of this review is to provide an overview of the use of biological materials in the augmentation of the anal sphincter either as part of an overlapping sphincter repair (OSR) or anal bulking procedure.

**Methods:**

A systematic search of PubMed was conducted using the search terms “*anal bulking agents,”* “*anal sphincter repair,”* or “*overlapping sphincter repair*.” Five studies using biological material as part of an overlapping sphincter repair (OSR) or as an anal bulking agent were identified.

**Results:**

122 patients underwent anal bulking with a biological material. Anorectal physiology was conducted in 27 patients and demonstrated deterioration in maximum resting pressure, and no significant change in maximum squeeze increment. Quality of life scores (QoLs) demonstrated improvements at 6 weeks and 6 months, but this had deteriorated at 12 months of follow up. Biological material was used in 23 patients to carry out an anal encirclement procedure. Improvements in QoLs were observed in patients undergoing OSR as well as anal encirclement using biological material. Incontinence episodes decreased to an average of one per week from 8 to 10 preoperatively.

**Conclusion:**

Sphincter encirclement with biological material has demonstrated improvements in continence and QoLs in the short term compared to traditional repair alone. Long-term studies are necessary to determine if this effect is sustained. As an anal bulking agent the benefits are short-term.

## Introduction

Fecal incontinence (FI) affects between 1 and 10% of adults in varying degrees. Current epidemiological studies have shown that up to 1% of adults have regular episodes of FI that adversely impacts on their quality of life (1). Treatment modalities vary from conservative, with the use of anti-diarrheal medications such as loperamide and codeine, to non-operative interventions such biofeedback strategies, to surgical management. Surgical options are usually indicated when continence is affected secondary to an anatomic disruption, such as a sphincter weakness or defect (2). Patients who have a history of colorectal surgery (dilatation), obstetric sphincter injury, or pelvic irradiation are also prone to fecal seepage and soiling (3).

The most common surgical procedure performed for the direct repair of an anatomic sphincter defect for FI is an overlapping sphincter repair (OSR) (4). Repairing the ends of the sphincter in an “overlapping” fashion has been shown to have slightly better results in comparison to a direct end-to-end repair (4). OSR is ideal for an isolated single defect (often obstetric trauma related) and bulking is reported ideal for a reduced hemorrhoidal cushion: anal canal ratio (5, 6). An anal encirclement, usually referred to as a Thiersch procedure, is the insertion of a prosthesis around the anal sphincter, thus narrowing the anal opening and is performed in patients with rectal prolapse who have high operative risk and/or extreme old age (7). Originally carried out using a silver wire, but due to ulcers and other complications, newer sutures are used including nylon, Dacron, Silastic, Teflon, and silicon rubber materials have been described.

Passive FI results in fecal leakage and is more likely to be due to internal sphincter damage (8). This can occur during childbirth or as a complication from anal surgery, particularly following a lateral sphincterotomy or hemorrhoidectomy (9–11).

Injecting bulking agents into the anal sphincter complex is a relatively new modality for patients with passive FI or mild–moderate incontinence (2). It is proposed that the bulking agents act to augment the anal cushions, thus providing an improved seal and therefore increasing the anal zone pressure (12). Furthermore, bulking agents are believed to improve anal canal symmetry and again, improve anal canal sealing (12). Neuromodulation techniques have recently become the reference standard for FI, but concerns persist regarding long-term effectiveness, costs, and complications arising from implanted devices. Sacral nerve stimulation has been used increasingly for FI in patients with external anal sphincter defects, but it is invasive and expensive (13).

The aim of this review is to provide an overview of the biological materials that have been used to augment an overlapping sphincter repair and as a bulking agent (14).

## Methods

A systematic search of PubMed was conducted using the search terms “*anal bulking agents,” “anal sphincter repair” or “overlapping sphincter repair*.” Titles, abstracts, and finally full texts were analyzed for studies reporting on the use of biological mesh. Inclusion criteria were studies that utilized biological material for either sphincter repair or as a bulking agent. Studies were excluded if only synthetic material was used. Furthermore, studies on patients under the age of 18 were excluded as well as non-English language studies, technical tips or duplicates series from the same research group. Overall, the search yielded five studies for analysis after the exclusion of review articles. The study characteristics are presented (Table 1).

**Table 1:** **Biologic materials augmenting the anal sphincter for the treatment of fecal incontinence**.

Reference	Study design	No. of patients	Age	Sex (M:F)	Patient characteristics	Material used	Follow-up (months)	Outcome	Complications	LofE
**ANAL BULKING**										
Kumar et al. (16)	Case series	17	NS	5:12	Idiopathic fecal incontinence secondary to weakness of the internal anal sphincter:9 incontinent following hemorrhoidectomy: 3. Following an internal sphincterotomy. obstetric injury: 2. Following surgical treatment for fistula in ano: 1	Glutaraldehyde cross-linked (GAX) collagen	8 (4–12)	Mean resting pressures: preop: mean 30 cm H_2_O, Postop: 45 cm H_2_O	None	4
								Squeeze pressures: were not significantly different before Preop: 125 cm H_2_O, Postop: 130 cm H_2_O		

Maeda et al. (17)	RCT (pilot)	10	68 (45–79) median	1:9	Passive fecal incontinence due to internal anal sphincter (IAS) dysfunction	Bulkamid™: 5 Permacol™: 5	19 (14–22) 1 lost to f/u	Median St Mark’s incontinence score: baseline: 16 (11–24), 6 weeks: 14 (3–18), 6 months:15 (8–22)	None Improved at 6/52 but deteriorated at 6/12, No difference between Bulkamid™ and Permacol™	2
								Maximum resting pressure (cm H_2_O): baseline:28 (15–58), 6 weeks: 27 (19–56), 6 months: 22 (10–38) (*P* < 0.05, baseline vs. 6 months)		
								Median maximum squeeze increment: baseline: 36 (16–109), 6 weeks: 44 (13–102), 6 months: 38 (15–186) (*P* < 0.32, baseline vs. 6 months)		
								FIQL: (preop vs. postop), Lifestyle score: median 3.10–3.50 (*P* < 0.05), Coping: 2.36–2.75 (*P* < 0.05). Depression: 2.42–3.70 (*P* < 0.005). Embarrassment: 1.67–1.84 (*P* < 0.05). SF-36: preop: median 29, Postop: 100		

Maslekar et al. (15)	Case Series	100	61 (36–82) mean	30:70	Fecal incontinence: Idiopathic 70% Traumatic 15% Neuropathic 10% Mixed 5%	Permacol^®^	Min 36, 10 lost to f/U	Preop: median squeeze pressures 54.7 (21.1–112.2)	None	4
								Median resting pressures 40.4 (18.1–89.9)		
								CCFIS Preop: median 14 (9–18), 6 weeks: 6 (5–14), 36 months: 8 (6–12)		
								38% repeat injection after first injection at a median of 12 months (4–16 months). 15% required an additional injection at a median of 18 months (14–20 months) from first injection.		
**SPHINCTER REPAIR**										
Zutshi et al. (18)	Case Control (age matched)	10 Permacol^®^ + 10 OSR	Group 1: 61.6 (46.7–76.3). Group 2: 64.7 (43.7–72.6)	0:10	Fecal incontinence (moderate to severe) with sphincter defect	Group 1: OSR with Permacol^®^	Group 1: 12.6 (8.8–22.7)	Group 1: FISI preop: 33.5 (21–46) Postop:14 (0–43) *P* = 0.02 CCFIS preop: 15 (11–18) Postop:10 (0–17) *P* = 0.005	None	4
						Group 2: traditional OSR	Group 2: 17 (9.6–34.4)			
								FIQL improved in coping/behavior, *P* = 0.02, and embarrassment, *P* = 0.01		
								Patient satisfaction with procedure: group 1 = 80% vs. Group 2 = 40%		

Zutshi et al. (19)	Case series	13	68.6 (59–79)	NS	Fecal incontinence with anal sphincter defect	Anal encirclement with sphincter repair Surgisis™	16.3 (6–24) mean	Mean anal resting pressure	None	4
								Preop: 33.23 (20–60.75) mmHg		
								Mean squeeze pressure: 70.09 (46.75–99) mmHg		
								Preop: FISI Preop: 39.22 (±16.1). Postop: 9.66 (±1.9)		
								Wexner Preop:18.33 (±5.04). Postop: 7.5 (±4.94)		
								Decrease of incontinence episodes from 8 to 10/week to 1/week		

*FISI, Fecal Incontinence Severity Index; CCFIS, Cleveland Clinic Fecal Incontinence Score; FIQL, Fecal Incontinence Quality of Life scale; NS, not specified*.

## Results

### Anal Bulking

There were three studies (a case series of 100 patients, a case series with 17 patients and an RCT pilot study with 10 patients) (15–17) where patients underwent anal bulking. Of these, 122 patients received a biological material as a bulking agent. Overall, 105 patients received additionally cross-linked porcine dermal collagen paste (Permacol™) and a further 17 received Glutaraldehyde cross-linked (GAX) collagen, a highly purified bovine dermal collagen. Anorectal physiology was carried out in only 27 patients and demonstrated deterioration in maximum resting pressure, and no significant change in maximum squeeze increment.

St Mark’s incontinence score was used in the RCT of 10 patients and demonstrated an improvement at 6 weeks, but deteriorated at 6-month follow up (17). The SF-36 quality of life scale showed a significant improvement in the role of a physical score from 29 to 100 after the injections. Similarly the Cleveland Clinic Florida Incontinence Score (CCFIS) was used in the second study on 100 patients and demonstrated an improvement in scores at 6 weeks and 6 months, but this had deteriorated at 12-month follow up (15). Another study carried out anorectal physiology in 17 patients but did not use any scoring system (16). There were no complications reported in any of the studies.

### Sphincter Repair

One study with 10 patients used an additionally cross-linked porcine dermal collagen (Permacol™) to augment an OSR (18). A traditional OSR dissection was carried out and the Permacol™ implant sutured to the under surface of the two muscle arms. Another study with 13 patients used porcine intestinal submucosa (Surgisis©) to carry out an anal encirclement procedure (19). A tunnel was created around the anal canal through which the Surgisis©graft was passed through and tightened. Validated incontinence scores such as the Fecal Incontinence Severity Index (FISI), Wexner, CCFIS and Fecal Incontinence Quality of Life (FIQL) have been used to assess outcome. Improvements in FISI, CCFIS and two sub scales of FIQL (coping/behavior and embarrassment) were observed in the group of patients undergoing OSR (18). Furthermore, the FISI, Wexner score and all components of FIQL score (lifestyle, coping/behavior, depression and embarrassment) showed an improvement following anal encirclement using biological material (19). Incontinence episodes decreased to an average of one per week from 8 to 10 preoperatively and there were no complications reported.

## Discussion/Summary

There are many different methods to improve symptoms of FI and despite limited evidence; they have been adopted to varying degrees. Agents such as autologous fat were first injected into the anal canal to create bulk and resistance in 1995 (20). Since then, other agents have been injected into the anal canal including polytetrafluoroethylene, carbon-coated zirconium oxide beads (Durasphere) and dextranomer microspheres in non-animal stabilized hyaluronic acid gel (NASHA) hydrogel cross-linked with polyacrylamide to name but a few (21) biologic materials are relatively new in comparison and their use is becoming more widespread. However, as the literature search above demonstrates, the evidence advocating their use is limited. There is only one pilot study for a controlled trial that randomized five patients to receive a biological injectable agent and five to receive a synthetic injectable agent (17) and the remainder are small case series of low level evidence. Short-term outcomes are promising and show some improvement in incontinence scores, but only half the studies used patient reported outcomes in the form of the FIQL scale. With regards to anal bulking with biologic material; there is an initial improvement but this improvement does not appear to be sustained at 12-month follow up. It was postulated that the operative technique may play an important role as biological agents injected sub mucosally, proximal to the dentate line, had better outcomes than agents injected via the trans-sphincteric route (22). Long-term follow up data is required in the form of prospective controlled trials. Outcomes are poor in comparison to NASHA DX, which has high quality RCT evidence to support its use. Anal bulking may offer some improvement to a select subgroup of patients, but NASHA DX should be the agent of choice. The use of biologics, especially given the cost, cannot be justified.

Anal encirclement with a prosthetic sling of silicone, which aims to reinforce the repair and the damaged external anal sphincter muscle has been demonstrated to have similar outcomes to alternative surgical procedures, although a high risk of breakage and fecal impaction (23, 24). Only one study was identified using biological material for anal encirclement and did not report any complications. In the small group analyzed, patients benefited from augmentation of the external anal sphincter, but long-term follow up is required to determine if this benefit is sustained (19).

Finally, OSR using biological tissue does not appear add morbidity. Sphincter augmentation has been in significant improvement in continence and quality of life scores (QoLs) compared with the preoperative scores in the short term over traditional repair (18). Long-term studies are necessary to determine if this effect is sustained.

## Conflict of Interest Statement

The authors declare that the research was conducted in the absence of any commercial or financial relationships that could be construed as a potential conflict of interest.

## Author Note

The data in this paper has, in part, been presented as a poster at the following meeting: Digestive Disorders Federation, ExCel London, June 2015.

## Appendix

### BioMesh Study Group

Ferdinand Köckerling (Chairman), Stavros Antoniou, René Fortelny, Frank A. Granderath, Markus Heiss, Franz Mayer, Marc Miserez, Agneta Montgomery, Salvador Morales-Conde, Filip Muysoms, Alexander Petter-Puchner, Rudolph Pointner, Neil Smart, Marciej Smietanski, and Bernd Stechemesser.

### Aim

The BioMesh Study Group has set itself the task of identifying how best to use biological meshes for the various indications. The first step toward achieving that goal is to compile systematic reviews of the different indications on the basis of the existing literature. The available literature sources will be evaluated in accordance with the Oxford Centre for Evidence-based Medicine-Levels of Evidence (March 2009). Next, based on the review findings, corresponding Statements and Recommendations are to be formulated in a Consensus Conference for the use of biological meshes for the different indications. The findings of the Consensus Conference are then to be summarized for a joint publication. This present publication is part of the project undertaken by the BioMesh Study Group.
